# Multi-Omic Approaches in Colorectal Cancer beyond Genomic Data

**DOI:** 10.3390/jpm12020128

**Published:** 2022-01-18

**Authors:** Emilia Sardo, Stefania Napolitano, Carminia Maria Della Corte, Davide Ciardiello, Antonio Raucci, Gianluca Arrichiello, Teresa Troiani, Fortunato Ciardiello, Erika Martinelli, Giulia Martini

**Affiliations:** 1 Oncologia Medica, Dipartimento di Medicina di Precisione, Università degli Studi della Campania “L. Vanvitelli”, 80131 Napoli, Italy; emisardo@gmail.com (E.S.); stefania.napolitano@unicampania.it (S.N.); carminiamaria.dellacorte@unicampania.it (C.M.D.C.); gianluca.arrichiello@unicampania.it (G.A.); teresa.troiani@unicampania.it (T.T.); fortunato.ciardiello@unicampania.it (F.C.); erika.martinelli@unicampania.it (E.M.); 2 Oncologia Medica, Fondazione IRCCS Casa Sollievo della Sofferenza, 71013 San Giovanni Rotondo, Italy; davide.ciardiello@unicampania.it; 3 U.O.C. Radiologia Generale e Pronto Soccorso, AORN Antonio Cardarelli, 80131 Napoli, Italy; antonio.raucci@gmail.com

**Keywords:** omics, colorectal cancer, biomarkers, multiparametric approach

## Abstract

Colorectal cancer (CRC) is one of the most frequent tumours and one of the major causes of morbidity and mortality globally. Its incidence has increased in recent years and could be linked to unhealthy dietary habits combined with environmental and hereditary factors, which can lead to genetic and epigenetic changes and induce tumour development. The model of CRC progression has always been based on a genomic, parametric, static and complex approach involving oncogenes and tumour suppressor genes. Recent advances in omics sciences have sought a paradigm shift to a multiparametric, immunological-stromal, and dynamic approach for a better understanding of carcinogenesis and tumour heterogeneity. In the present paper, we review the most important preclinical and clinical data and present recent discoveries in the field of transcriptomics, proteomics, metagenomics and radiomics in CRC disease.

## 1. Introduction

Colorectal cancer (CRC) is one of the most frequent tumours and one of the major causes of morbidity and mortality globally. Its incidence has increased in recent years, being the third most common worldwide with approximately one million new cases per year, particularly in developed countries [1]. This dramatic increase could be linked to risk factors such as unhealthy dietary habits, stress, smoking, and a sedentary lifestyle that, combined with environmental and hereditary factors [2], can lead to genetic and epigenetic changes in normal epithelial cells and induce tumour development. The polyp cancer progression sequence model described by Fearon and Vogelstein is considered a parametric, static and complex model involving oncogenes (e.g., Kirsten rat sarcoma viral oncogene (KRAS), Neuroblastoma RAS viral oncogene homolog (NRAS), V-raf murine sarcoma viral oncogene homolog B1 (BRAF), and phosphatidylinositol-4,5-bisphosphate 3-kinase, catalytic subunit alpha (PIK3CA)), tumour suppressor genes (e.g., tumour protein P53 (Tp53), adenomatous polyposis coli (APC), phosphatase and tensin homolog (PTEN)) and pathognomonic signalling pathways that modulate cell differentiation, proliferation and apoptosis in the CRC: Wnt/β-cadherin, epidermal growth factor receptor (EGFR), mitogen-activated protein kinase (MAPK), transforming growth factor beta (TGF-β) and phosphoinositide 3-kinase (PI3K) [3]. Despite multiple efforts to better understand tumorigenesis, the lack of new biomarkers and tumour heterogeneity present many unclarified challenges. Since the human genome project, omics sciences have revolutionised the study of CRC. Transcriptomics, proteomics, metagenomics, metabolomics and radiomics contribute to a paradigm shift towards a multiparametric, dynamic, immunological and stromal model that allows for a better understanding of CRC development as well as its classification into different molecular subtypes for patient stratification and the development of new biomarkers and targeted therapies. This review highlights the contributions of transcriptomics, proteomics, metagenomics and radiomics over the last few years in building a multi-omics model for a better understanding of tumour development and heterogeneity to ensure optimal treatment of CRC.

## 2. Genomics

CRC is one of the first tumours to be molecularly profiled and multiple genes and pathways involved in tumorigenesis have been identified but only a limited number are recurrently mutated in a large proportion of tumours that play a crucial role. Molecular defects can be of two types: alterations leading to a new or enhanced function of oncogenes and disorders causing loss of function of tumour suppressor genes. The switch of cellular genes to oncogenic variants as well as the inactivation of tumour suppressor genes may be the result of point mutations or rearrangements that modify the structure or function of these genes, as well as chromosomal amplifications that affect regulated gene expression. Genes with defects in key functions in tumour initiation, progression and/or maintenance are called driver genes [4,5]. The Cancer Genome Atlas (TCGA), through a multidimensional analysis of 276 samples, has identified 32 somatic recurrently mutated genes, of which APC, TP53, KRAS, NRAS, PIK3CA, FBXW7, SMAD4, TCF7L2 CTNNB1, SMAD2, FAM123B (also known as WTX) and SOX9 were the most frequently mutated [6].

The Ras gene family encodes for cytoplasmic proteins with GTPase activity: H-ras, K-ras and N-ras. V-Ki-ras2 Kirsten rat sarcoma viral oncogene homolog (KRAS) is mutated in 40% of CRC cases, mostly in exon 2, (codons 12 and 13) and less frequently in exon 3 (codon 61) and exon 4 (codon 146), followed by NRAS, which is present in approximately 3–5% of CRC and can be found in exon 3 (codon 61) and exon 2 (codons 12, 13) [7,8]. Ras mutations can be detected by real-time PCR (RT-PCR), BEAMing, Next-generation sequence (NGS) and Sanger sequencing but recently the use of liquid biopsy (LB) has been implemented as an emerging tool with a concordance between plasma and tissue of approximately 90% [9,10]. One of the particularities of this genetic alteration is that acquired RAS mutations are maintained throughout carcinogenesis, as can be seen by the almost perfect concordance of RAS mutation status in primary and metastatic colorectal cancer. On the other hand, the V-raf murine sarcoma viral oncogene homolog B1 (*BRAF*) gene is mutated in 10–15% of patients with CRC, it is found in exon 15 and around 80% corresponds to the T17991 transversal mutation causing the V600E amino acid substitution, while the other 20% corresponds to a wide variable range of missense mutations all residing in the G-loop glycines in exon 11 or in exon 15 near V600 [11,12,13]. The V600E substitution results in constitutive MAPK phosphorylation and subsequent RAF-mitogen-activated protein kinase (MEK)-extracellular signal-regulated kinase (ERK) signal transduction. RAS and BRAF mutations are both associated with a poor prognosis and are mutually exclusive, lending additional support to the hypothesis that an activating mutation in either gene is sufficient to promote tumorigenesis by increasing MAPK signalling and is also related to MSI tumours. In addition to its prognostic role, multiple studies have investigated its potential predictive role in the development of target therapies to help optimise the treatment of metastatic colorectal cancer.

Amplification in the human epidermal growth factor receptor 2 (HER2) gene (ERB2) has been described in approximately 3–4% of CRC patients, and is highly enriched in KRAS wild-type (6–8%) and MSI-H patients [14,15]. Very recent studies have described the sensitivity of HER2 amplified tumours to anti-EGFR therapy [16,17,18]. Also, other genes such as ALK, ROS, and NTRK occur in less than <1.5%. Fusions in NTRK genes are more commonly detected in non-Lynch syndrome/MSI-H tumours as well as in wild-type BRAF, KRAS, and NRAS tumours. However, it remains unclear precisely which CRC population should be tested. Molecularly driven therapy with the selective TRK inhibitor larotrectinib and multikinase inhibitors with activity against these fusion proteins has been tested in selected populations with NTRK fusions. Finally, RET fusions have been found in a small fraction of patients with CRC (<1%), predominantly on the right side, RAS and BRAF wild-type tumours, and carry a worse prognosis compared to patients without RET fusions [19,20,21]. 

Although several pathways are involved in CRC carcinogenesis, TCGA provided data that 93% of tumours showed alterations in the WNT/B-catenin pathway through inactivation of the APC gene or active mutations of the CTNNB1 gene as well as alteration of negative regulatory genes such as ARID1A and FAM123B. Genetic alterations in the RAS-MAPK and PI3K pathways were reported as the second most common, not only due to overexpression of the insulin-like growth factor 2 (IGF2) gene, but also due to mutations in PIK3CA and in the Ras gene family. Another pathway deregulated in CRC is the TGF-B pathway, where genomic alterations were found in the TGFBR1, SMAD4-SMAD3-SMAD2 and ACVR2A genes [6,22,23,24].

Defects in DNA repair genes can occur due to germline mutations, somatic mutations or gene silencing leading to biallelic inactivation of these genes. The DNA mismatch machinery consists of four proteins (MSH2, MSH6, MLH1 and PMS2) so that when a loss of function of any of them occurs, a cascade of favourable events is triggered in the development of CRC [25,26]. Immunohistochemistry (IHC) and genomic sequencing tools can be used for diagnosis; IHC detects the loss or absence of some of the proteins, while PCR can analyse the corresponding loci (BAT-25, BAT-26, DS2S123, D5S346 and D17S250) [27]. Microsatellite instability status occurs in 5% of metastatic colon tumours and 15% of primary tumours and may have predictive and prognostic value, respectively. The combination and sum of these alterations lead to a genetic classification of CRC carcinogenesis, and two phenotypes can be identified: [1] The *CIN phenotype* (65–70%) is related to defects in chromosomal segregation, telomeric stability and mutations in APC, KRAS and TP53; [2] *MSI phenotype* (15%), which are hypermutated as a result of a defective DNA mismatch repair (MMR) system. This categorisation provides a linear model of carcinogenesis that has raised several unresolved questions about tumour heterogeneity.

## 3. Transcriptomics

**Figure 1:**
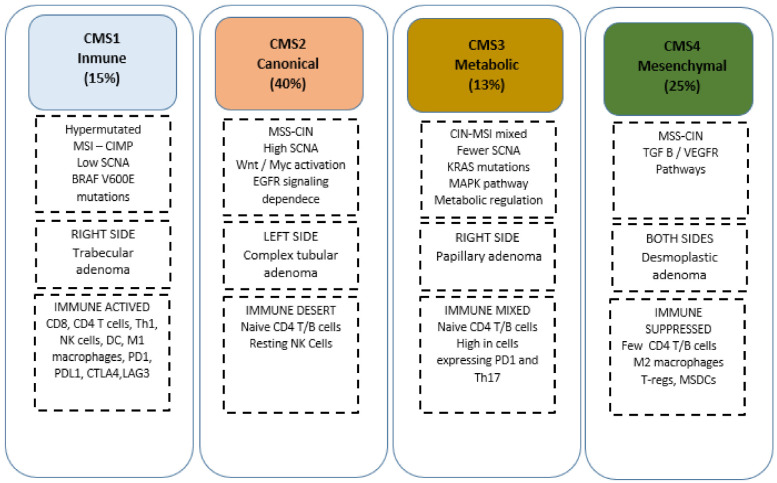
CMS classification. CIN, chromosomal instability; CMS, consensus molecular subtypes; DC, dendritic cell; EGFR, epidermal growth factor receptor; MSDC, myeloid-derived suppressor cells; MSI, microsatellite instability; MSS, microsatellite stability; NK, natural killer; PD-1, programmed cell death protein 1; TGF-β, transforming growth factor beta; Tregs, regulatory T cells; LAG3, lymphocyte activating 3; Th17, lymphocyte T helper 17.

The information present in the DNA and its genetic and epigenetic changes are expressed by transcription, detailing the most precise activity of a cell at that moment and its close relationship with the tumoral phenotype and its subsequent clinical behaviour. From the linear genetic model of carcinogenesis pathways to the advancement of technologies to study the transcriptome, multiple attempts have been made to molecularly characterise CRC. In the absence of a gold standard for molecular analysis of CRC, in 2015 a group of experts evaluated in an exhaustive and methodological way all the molecular classifications of CRC obtained through different approaches, to achieve an integrative set of samples that could resolve the inconsistencies of the pre-existing classifications and unify all the existing data not only on gene expression but also at the level of mutational burden, copy number, microRNAs, methylations and proteins in order to achieve four molecular subtypes (CMSs), being the clearest and most consistent classification in use up to date [28]. The main characteristics of each subtype are summarised as follows: at the genomic aberration level, CMS1 (~15%) is characterised by a hypermutated and generalised hypermethylated status, with low Somatic Copy Number Alteration (SCNA) in concordance with TCGA data as well as overexpression of proteins involved in DNA repair. On the other hand, CMS2 (~40%) and CMS4 (~25%) are characterised by high chromosomal instability (CIN) and high SCNA. Meanwhile, CMS3 (~13%) has a mixed pattern with few SCNAs but also a state of moderate hypermutation. Despite attempts to identify specific mutations in each subgroup, we were only able to identify a higher presence of BRAF mutations in CMS1 and KRAS mutations in CMS3, but without achieving a distinctive pattern. Finally, at the level of gene expression, CMS1 was characterised by the presence of genes associated with immune infiltration and with activation of immune evasion pathways, while CMS2 was closely associated with the Wnt/Myc pathways. By contrast, CMS3 showed an enhancement of multiple metabolic signatures and CMS4 a pronounced upregulation of genes associated with epithelial to mesenchymal transition and signatures linked to the activation of TGF-β signalling. Moreover, multiple subsequent investigations have tried to relate these subgroups to different events (clinical-pathological relationship, immune–microenvironment interaction, and prognosis–treatment association] to achieve a possible refinement of the classification (Figure 1). 

The clinical-histopathological variables through the CMSs are associated with different histological types of the precursor adenoma; trabecular-mucinous, complex tubular structure, papillary and desmoplastic reaction for CMS1, CMS2, CMS3 and CMS4, respectively [28,29]. In addition, a relationship with their localisation has also been observed; CMS1 is predominantly found on the right side and CMS2 on the left side and may be correlated to their respective mutational characteristics [30]. Another clinical feature observed has been the relationship between CMSs and the different stages of CRC, with CMS4 being the most frequently found in advanced stages. However, converting these relationships into a gold-standard pattern is complicated by the intratumoral heterogeneity of CMSs in the same sample observed in different studies. This pronounced heterogeneity is probably explained by gene expression variations between the different regions of the tumour and in association with the components of the tumour microenvironment (immune-stromal content). The different immuno-stromal phenotypes of CRC can be directly related to genomic events and the production of immunogenic peptides as well as the density of CTL infiltration [31]. This leads to two major immuno-stromal phenotypes [1]; Highly immunogenic: hypermutated tumours with DNA repair defects, high infiltration of Cytolytic T Lymphocytes (CTLs) and Lymphocyte T helper 1 (Th1), high expression of CTL-associated antigen 4 (CTLA4), programmed cell death protein 1 (PD1), PD1 ligand 1 (PDL1), and indoleamine 2,3 dioxygenase 1 (IDO1] [2]; Inflamed: high infiltration of regulatory T cells (T-reg) cells, myeloid-derived suppressor cells (MDSCs], intimately related to TGF-β and genes encoding cytokines IL-23 and IL-17 [32,33]; more recently, Thorsson et al. [34] presented a possible global immune classification of solid tumours based on the transcriptomic profiles where six groups were detailed (Table 1). 

The application of these phenotypes across CMSs taking into account their genomic and transcriptomic framework can establish that CMS1 would be immune active, while CMS4 could be related to the inflammatory or immunosuppressed pattern; in turn, CMS2 is considered immune desert and CMS3 immune mixed. The prognostic value of CMS subgroups has been extensively studied over the last few years but Galon and colleagues [35,36,37] were among the first to highlight the relevance of immunologic phenotypes in the prognosis of early-stage CRC, describing that high lymphocyte infiltration, especially of Th1 CTLs and interferon gamma (IFNγ), correlates with positive overall survival (OS) and disease-free survival (DFS), and higher levels of interleukin (IL 17) and Th17 are associated with worse outcomes, which may be linked to their ability to develop pro-metastatic immune evasive mechanisms. On the other hand, the predictive value in early stages was demonstrated only by a retrospective analysis of the MOSAIC trial where CMS2 patients benefited from the use of Oxaliplatin as adjuvant treatment. In the metastatic setting, retrospective analyses of clinical trials such as CALGB-80045 and FIRE 3 showed that CMS2 was associated with a positive OS, while CMS4 and CMS1 were associated with poor and intermediate OS, respectively [38,39]. The CALGB 80045 study demonstrated that the use of Bevacizumab in CMS1 was associated with better OS compared to the use of Cetuximab and that CMS2 showed a prolonged OS under treatment with Cetuximab, which could be explained by the intimate relationship of CMS1 with BRAF mutations and an MSI state. In addition, immunogenic subtypes, especially CMS1, have a greater tendency to respond to immunotherapy than immunosuppressed tumours. Despite all these developments, further efforts are needed to refine the CMS classification to assess its predictive role and for the development of optimal therapies.

## 4. Proteomics

Proteins represent key actors in several biological processes and their expression could be altered by the presence of gene mutations. The proteome is the functional translation of the genome, as well as a useful source of potential biomarkers. Protein biomarkers are notably up- or downregulated in the cancer proteome as compared to the normal proteome; for this reason, in recent years, proteomics research has focused on identifying differential expression characteristics between normal and cancer cells; detecting proteins involved in cancer formation and progression as well as observing the effects of protein perturbation or modification to provide new classification tools such as possible diagnostic, prognostic and predictive biomarkers in CRC. 

Several preclinical studies have identified the proteome of CRC cell lines and murine models, to underline the biological changes that affect CRC disease. CRC cell line secretome has been studied as a part of a large analysis of different solid tumour cell lines in which 4584 non-redundant proteins were identified and 30% of these were found in a ubiquitous manner along with different tumour types. On the other hand, 109 proteins were found only in CRC cells, thus demonstrating specificity for CRC and potentially being considered as biomarkers of disease [40]. A lot of other differences in in vitro studies showed the presence of different proteomics biomarkers, also in correlation with treatment, such as the study by Boisvert et al., in which the authors found that DNA damage could change subcellular proteomic localisation by performing a proteomics analysis [41]. Proteomics studies also involved engineered murine models. Interestingly, Zhu and colleagues utilised APC−/+ mouse models and identified 27 up-regulated proteins in tumour tissue, compared to the normal one. Another group found biomarkers such as MCM4, S100A9 and CHI3L1 in CRC proteasome proximal fluids of conditional APC knockout mice, compared to healthy mice with normal mucosa [42]. Several proteomics studies have been performed in a clinical setting using biological samples such as blood, stools and tissue, with the same aim to identify putative biomarkers of CRC disease. However, even if a lot of effort has been made over the last 20 years in the field of proteomics, predictive proteomic biomarkers of response to treatment have not yet been defined. In fact, all the analyses have been conducted in small cohorts and have not provided the expected results since a plethora of biomarkers have been identified but none of these similarities between the different studies. Moreover, none of the abovementioned biomarkers found in a “discovery phase” have reached a “validation phase”, thus precluding their use in a clinical setting [43,44,45,46,47] (Table 2). Integration of proteomics with transcriptomic and genomic data and the implementation of technologies such as mass spectrometry assays could overcome the heterogeneity of proteomics biomarkers.

## 5. Metagenomics

Metagenomics is the study of a microbiota community. The microbiota is the set of microorganisms (bacteria, viruses, fungi, protozoa, worms and archaea) that inhabit the human body. This science allows the discovery of microbial communities in their complex natural environment and their relationship with the host using techniques based on sequence divergences of the small subunit ribosomal RNA (16S rRNA) as NGS, denaturing gradient gel electrophoresis (DGGE), fluorescence in situ hybridisation (FISH), terminal restriction fragment length polymorphism (T-RFLP) and DNA microarrays [48]. The Human Microbiota project emerged in the 2000s with the aim of characterising the human microbiome with more precision in order to determine the intrinsic relationship with diseases and to provide a standardised data source. Both metagenomics and HMP allowed the development of the gut microbiota profiling that represents 29% of the human microbiota and is mostly composed of prokaryotic microorganisms that maintain a dynamic and homeostatic symbiotic relationship with the host supporting a robust immune and nutritional system [49,50]. The disruption of this homeostatic process leads to the development of multiple diseases such as inflammatory bowel disease (IBD) and CRC. Initial evidence for host–microbiota interactions in CRC emerged in 1969 with the publication of Vivienne Aries et al. and in 1975 when it was shown that the carcinogen dimethylhydrazine triggered significantly less colonic tumorigenesis in germ-free rats than in those with gut microbiota [51], but over time, multiple studies have also demonstrated that pro-carcinogenic microorganisms can influence cell proliferation, genomic instability and the tumour microenvironment of CRC [50,52,53,54]. Enterotoxigenic *Bacteroides fragilis* (ETBF) secretes B-fragilis toxin (BFT) that binds to E-cadherin, allowing its translocation to B-cadherin and the subsequent activation of the proto-oncogene c-Myc and therefore the cell proliferation of the colonic epithelium; by a similar mechanism, *Fusobacterium nucleatum* binds to E-cadherin through FadA, activating the Wnt/B-cadherin pathway, while *Escherichia coli* (EC) releases the genotoxin colibactin (pks+), which causes senescent cells to secrete growth factors. Furthermore, in vitro studies have shown that the genotoxin colibactin pks+ and *Enterococcus faecalis* alkylates DNA, producing double-strand breaks, aneuploidy and microsatellite instability [55]; by contrast, ETBF can induce DNA damage by stimulating inflammation and a pro-oxidant microenvironment through the expression of the spermine oxidase enzyme (SMO). Moreover, the relationship between the microbiota and the immune system has allowed endogenous pathogens to interfere in the tumour microenvironment by activating pro-tumorigenic immune responses like ETBF increase in mice models the T-Th17 that is generally associated with worse prognosis in CRC and Fusobacterium nucleatum use Fad 2 adhesine binding to the T cell immunoreceptor with immunoglobulin and immunoreceptor tyrosine-based inhibitory motif domains (TIGIT) to silence the tumour-killing capabilities of cytotoxic immune cells, among other mechanisms described [56,57]. Therapeutic treatment of the gut microbiota has been the focus of recent studies (Refs. [58,59]); antibiotics, microbiota transplantation, vaccines and immunotherapy have been some of the therapies proposed but have not yet been enough to stop the development of CRC. The need for large, international studies with prospective and longitudinal sampling and a more focused study of colorectal cancer microbiota and emerging targeted therapies has led to the creation of two recent projects: the OPTIMISTICC project (Opportunity to investigate the microbiome’s impact on science and treatment in colorectal cancer) and MICROCOSM (Microbiome of colorectal cancer: a longitudinal study of mechanism), the results of which are awaited.

## 6. Radiomics

The progress to a multiparametric approach to CRC development is also closely linked to technological advances in medical imaging. Magnetic resonance imaging (MRI), computed tomography (CT) and fluorodeoxyglucose positron emission tomography (FDG-PET/CT) have diagnostic and prognostic value in all stages of CRC. Tumour location, volume, size and texture as well as FDG uptake represent important qualitative parameters that cannot reflect tumour heterogeneity. Since genomic profiling is essential for therapy in CRC, there have been several attempts to explore the potential role of radiomics in this context by developing radiogenomic models able to predict genomic mutations such as KRAS, BRAF and MSI status and enhance decision-making and patient outcomes. Lee et al. in 2016 attempted to predict KRAS status depending on C-reactive protein (CRP) levels using FDG-PET/CT; 179 patients of all stages were studied, 75% had normal CRP values and 25% had increased values. The maximum standardised uptake value (SUV_max_) relationship could only be demonstrated in KRAS mutated (KRASmt) patients with normal CRP values [60]. Years later, Arslan et al. also attempted to demonstrate by FDG-PET/CT the association of SUV_max_ with the coexistence of KRAS mutations in 83 patients with CRC; they found that SUVmax was higher in KRASmt patients than in wild-type patients (24.0+/−9.0 vs. 17.7+/−8.2) [61]. Chen et al. were also able to show, in a study of 74 patients, the association between radiomics and KRAS mutations using SUV_max_, 6 histograms and 40 textural indices [62]. Other works such as those of Oh et al. and Xu et al. were performed with MRI specifically for patients with rectal cancer. The first group was able to demonstrate that three radiomics features were significantly associated with KRAS mutation status, while Xu et al. observed that differences were higher in the KRASmt group [63,64]. Gonzalez Castro et al. in a study of 147 patients noted that grey-level pixels and spectral texture features CT-based radiomics can predict KRAS mutations [65]; these findings were supported by the group of Taguchi et al. [66]. On the other hand, two studies (Orner et al. and Krikelis et al.) that predict KRAS status by FDG-PET/CT failed to demonstrate a statistically significant relationship between SUV_max_ value and KRAS mutation status [67]. Similarly, Hong et al. also failed to find a significant relationship between MRI and KRAS mutation. By contrast, there is limited literature related to radiomics predicting BRAF mutations and MSI status in CRC [68]. Kawada et al. in 2012 with a retrospective study of 51 patients showed that KRAS/BRAF mutation correlated with higher SUV_max_ [69]. Similar results were found by Lei Yang et al. in 2018 that demonstrated that three CT radiomics feature signatures were significantly associated with KRAS/NRAS/BRAF mutations (*p* < 0.001) [70]; Negreros-Osuna et al. in 2020 show that BRAF mutation could be predicted by radiomics features [71]. Concerning MSI’s predicted status, two studies published in 2021 support the previous research findings of Pernicka et al. [72]. Both prospective and multicentre trials attempted to predict MSI status by CT using three models (clinical model, radiomics model and an integrated model); the clinical-radiomic model was in both cases the best predictor of the relationship with MSI status [73,74]. (Table 3 summarises the characteristics of radiomics studies.) 

The potential role of radiomics in the identification of new prognostic and predictive CRC biomarkers has been translated into the use of machine-learning algorithms to provide clinical information on innovative artificial intelligence (AI) models. These computational analyses have identified patterns that represent a diagnostic tool better than conventional radiomics models [75]. Recently, at ESMO 2021 Congress, an AI model to automatically detect MSI status in early CRC has been presented. AI integrated imaging has led to the identification of CRC on unstained tissue samples and subsequently to a dichotomic differential diagnosis between MSS and MSI status, even if with low specificity. Therefore, although AI imaging could represent an innovative approach to determine MSI status, larger studies are requested to further confirm these data [76].

Despite the high potential of radiomics, the small population numbers and lack of reproducibility are two major limitations. Future efforts with a better-defined population, combined (clinical-radiomic) models, and the definition of the most appropriate imaging method could better clarify the landscape for prospective studies and change clinical practice.

## 7. Conclusions

The development of omics sciences and their technologies has helped to understand the onset, progression and treatment of CRC in a more integrated manner. Genomic and transcriptomic profiling has the main role of establishing molecular subtypes with the corresponding stratification of patients to pave the way for personalised medicine. Both DNA and RNA are vectors of genetic information that encode proteins. Their study, through proteomics, allows the true functional interpretation of what happens at the cellular level in a given situation as well as its most crucial contribution to CRC in the identification of potential new biomarkers and targets for novel targeted therapies. The study of the microbiota provides a non-traditional tool with a future role in better understanding tumour biology, as does radiomics, which serves as a bridge between medical imaging and precision medicine, providing objective and accurate information that in the future will help to better understand intratumoral and intertumoral heterogeneity through the use of a non-invasive method. This multiparametric and holistic approach has provided short-term benefits through biomarkers and potential targets, but there is still a long way to see long-term benefits through early diagnosis and increased overall survival in CRC.

## Figures and Tables

**Table 1:** Thorsson et al.: global immune classification of solid tumours based on the transcriptomic profiles. Th1/Th2, lymphocyte T helper 1/2; TCR, T cell receptor; Th17; lymphocyte T helper 17; IFN-y, interferon y; TGF-β, transforming growth factor β.

C1 Wound Healing	Elevated expression of angiogenic genes High proliferation rate Low Th1/Th2 ratio related to the adaptive immune infiltrate.
C2 IFN-y dominant	High proliferation rate Highest intratumoral heterogeneity Macrophages M1/M2 polarisation CD8 T cell population TCR diversity.
C3 Inflammatory	Elevated Th17 and Th1 genesLow to moderate proliferation Lower levels of aneuploidy Higher somatic copy-number alterations
C4 Lymphocyte Depleted	Moderate cell proliferation and intratumoral heterogeneity Prominent macrophage signature with Th1 suppressed and a high M2 response
C5 Immunologically quiet	Lowest lymphocyte and highest macrophage, dominated by M2 Low rates of proliferation and heterogeneity.
C6 TGF- *β*	Mixed tumours with the highest TGF-b signature High lymphocytic infiltrate with a balanced Th1:Th2 ratio.

**Table 2:** Predictive proteomic biomarkers in clinical setting.

Biomarkers	Relevance	References
Apolipoprotein E 180 (APOE) Angiotensinogen (AGT) Vitamin D binding protein (DBP)	Survival outcomes in Bevacizumab-treated patients	Martin et al. (2014) [45]
Phosphorylated EGFR (pEGFR)	Response to Cetuximab	Katsila et al. (2014) [46]
Poly (C) binding protein 1 (PCBP1)	Oxaliplatin resistance	Guo et al. (2017) [47]
FAST Kinase Domains 2 (FASTKD2) Caldesmon 1 (CALD1) Carboxypeptidase A3 (CPA3) Receptor interacting serine/threonine-protein kinase 1 (RIPK1) Mast cell carboxypeptidase 4 (CPA3) Beta-1,3-galactosyltransferase 5 (B3GALT5) CD177 antigen (CD177 Dihydropyrimidine dehydrogenase (DPYD)	Response to neoadjuvant treatment (5-Fu/Capecitabine ± oxaliplatin) for rectal cancer	Chauvin et al. (2018) [48]
Plectin-1 (PLEC 1) Transketolase (TKT) Trifunctional enzyme subunit mitochondrial precursor (HADHA) Transgelin (TAGLN)	Response to 5-FU ± oxaliplatin	Croner et al. (2016) [47]
Fibrinogen B chain (FGB) Serpin B5–B9 Peroxiredoxin-4 (PRDX4) Cathepsin D (CTSD)	Response to 5-FU ± oxaliplatin	Repetto et al. (2017)

**Table 3:** Selected colorectal cancer radiogenomics studies on TC/MRI and FDG-PET/TC imaging. CT, computed tomography; MRI, magnetic resonance imaging; FDG-PET, fluorodeoxyglucose positron emission tomography; MSI, Microsatellite instability; SUVmax, Maximum standardized uptake value; KRAS_mt_, KRAS mutated; BRAF_mt_, BRAF mutated.

Year	Author	Complementary Imaging Method	Study	N	Study Population	Aim	Conclusion
2021	Cao et al. [75]	CT scan	R	502	Stage II–III	Prediction of MSI status	32 radiomics features show correlation with MSI status, the combined model (Clinical risk factors + radiomic features) is better to predict MSI status
2021	Li et al. [76]	CT scan	R	368		Prediction of MSI status	The combined model (tumour location + 8 radiomic features) can predict MSI status.
2020	Arslan et al. [63]	FDG-PET/CT	R	83	All stages	Prediction of KRAS status	SUV_max_ was higher in KRAS_mt_
2020	Oh et al. [65]	MRI	R	60	Rectal tumours All stages	Prediction of KRAS status	MRI imaging features (Skewness, médium texture) could predict KRAS_mt_
2020	Gonzalez-Castro et al. [67]	CT scan	R	47	All stages	Prediction of KRAS status	Radiomics features (texture in the tumour region + standard intensity) can predict the presence of the KRAS_mt_
2020	Negreros-Osuna [73]	CT scan	R	145	Stage IV	Prediction of BRAF status	Standard deviation (SD) and mean value of positive pixels (MPP) were lower in the BRAF_mt_ group.
2020	Cui et al. [74]	MRI	R	304	Rectal tumours All stages	Prediction of KRAS status	Seven radiomics features were moderated predicting KRAS status
2019	Chen et al. [64]	FDG-PET/CT	R	74	All stages	Prediction of KRAS status	KRAS_mt_ tumours had an increased value of SUV_max_
2019	Xu et al. [66]	MRI	R	158	Rectal Tumours stages II–III	Prediction of KRAS status	Six radiomic features were higher in the KRAS_mt_ group
2019	Taguchi et al. [68]	CT scan	R	40	Stage II–IV	Prediction of KRAS status	CT textures can predict the KRAS_mt_
2019	Pernicka et al. [74]	CT scan	R	198	Stage II–III	Prediction of MSI status	The combined model (Clinical + radiomic features) is better at predicting MSI
2018	Yang et al. [72]	CT scan	R	117	All Stages	Prediction of KRAS/NRAS/BRAF status	Three radiomics features could be useful for predicting KRAS_mt_/NRAS_mt_/BRAF_mt_
2017	Coner et al. [47]	FDG-PET/CT	R	55		Prediction of KRAS status	No significant association between KRAS gene mutation and SUV_max_, MTV, TLG and haematological parameters.
2016	Lee et al. [62]	FDG-PET/CT	P	179	All stages	Prediction of the KRAS status depending on CRP level	Higher SUVmax in KRAS_mt_ patients with normal CRP
2016	Lovinfosse et al.	FDG-PET/CT	R	151	All stages	Prediction of KRAS, NRAS, BRAF	No significant association between quantitative parameters and KRAS, NRAS, BRAF status
2015	Kawada et al. [69]	FDG-PET/CT	R	55	Stage IV	Prediction of KRAS status	SUV_max_ remained significantly associated with KRAS_mt_ in tumours larger than 10mm
2014	Krikelis et al. [67]	FDG-PET/CT	R	44	Stage IV	Prediction of KRAS status	No significant correlation between SUV_max_ values and KRAS_mt_
2013	Hong et al. [70]	MRI	R	29	Rectal Tumours Stages II–III	Prediction of KRAS status	No significant correlations between MRI parameters and KRAS_mt_
2012	Kawada et al. [69]	FDG-PET/CT	R	51	All stages	Prediction of KRAS-BRAF status	Higher FDG accumulation in patients with KRAS_mt_ and BRAF_mt_ and can be used to predict mutations
